# Catechol-*O*-methyltransferase *Val^158^Met* Genotype and Early-Life Family Adversity Interactively Affect Attention-Deficit Hyperactivity Symptoms Across Childhood

**DOI:** 10.3389/fgene.2020.00724

**Published:** 2020-07-10

**Authors:** Eyal Abraham, Marc A. Scott, Clancy Blair

**Affiliations:** ^1^Department of Psychiatry, Columbia University Vagelos College of Physicians and Surgeons, New York, NY, United States; ^2^Division of Translational Epidemiology, New York State Psychiatric Institute, New York, NY, United States; ^3^Department of Applied Psychology, New York University, New York, NY, United States; ^4^Department of Applied Statistics, Social Science, and Humanities, New York University, New York, NY, United States; ^5^Department of Population Health, New York University Grossman School of Medicine, New York, NY, United States

**Keywords:** COMT, ADHD, early-life adversity, parenting, socioeconomic risk, longitudinal studies, childhood

## Abstract

Attention-deficit hyperactivity disorder (ADHD) is among the most commonly diagnosed psychiatric disorders of childhood. The dopaminergic system has been shown to have substantial effects on its etiology, with both functional Catechol-*O*-methyltransferase (*COMT*) *Val^158^Met* genotype and early-life environmental adversity involved in the risk of inattention and hyperactivity/impulsivity symptoms. In this prospective longitudinal study, we examined for the first time the impact of proximal and distal early-life family adversity and *COMT Val^158^Met* polymorphism gene – both the direct and the interactive effects, on children’s ADHD symptoms across childhood. Data came from the Family Life Project, a prospective longitudinal study of 1,292 children and families in high poverty from birth to 11 years. In infancy, data regarding socioeconomic (SES)-risk-factors, observed-caregiving behaviors, and DNA genotyping were collected. In early and middle childhood teachers rated the occurrence and severity of the child’s ADHD symptoms. Multilevel growth curve models revealed independent effects of *COMT*, early-life SES-risk and negative caregiving on ADHD symptoms in early and middle childhood. Significant gene-environment interactions were found, indicating that overall, carriers of at least one *COMT^158^Met* allele were more sensitive to early-life adversity, showing higher inattention and hyperactivity/impulsivity symptoms severity in childhood when exposed to high SES-risk factors in infancy, compared to Val-Val carriers. Findings provide new insights into the complex etiology of ADHD and underline the need for further investigation of the neuronal mechanisms underlying gene-environment interactions. Findings might have implications for prevention and intervention strategies with a focus on early-life family relationships in genetically at-risk children.

## Introduction

Attention-deficit hyperactivity disorder (ADHD) is one of the most prevalent childhood-onset neurodevelopmental disorders, affecting approximately 7% of children and adolescents (Thomas et al., 2015). ADHD is composed of two correlated, yet distinct, symptom dimensions: inattention and hyperactivity/impulsivity, which may be associated with partially distinct neuropsychological mechanisms (American Psychiatric Association, 2013). If gone untreated or undiagnosed, ADHD may persist over the lifespan, can adversely influence the way the child develops cognitively, emotionally and socially, and places the child at a particular risk for developing other comorbid disorders as well as adult psychiatric disorders (Barkley, 2014).

ADHD is a complex and heterogeneous disorder, and its etiology is not yet completely understood (Nigg, 2012). Both genetic and environmental studies of ADHD implicate catecholamines in its etiology, mainly the neurotransmitter dopamine (DA). DA plays a role in modulating attention, response inhibition, cognitive processing, working memory, motivation, and reward; all of which are frequently impaired in ADHD (Solanto, 2002). A large body of human and animal studies indicates that early-life adversity leads to exaggerated catecholamine activity in PFC by blocking the extraneuronal catecholamine transporters on glia that clear the extrasynaptic space of catecholamines (Pruessner et al., 2004; Fogelman and Canli, 2019). Catecholamine actions impact the development of DA mesocortical pathways in the brain (Meaney et al., 2002; Weber et al., 2018), shift the brain’s functional architecture from the PFC and self-regulation to the amygdala and rapid reflexive-regulation (Arnsten, 2009), which, ultimately, increase impulsivity, social aggression (Parent and Meaney, 2008) and attention dysfunction (Francis and Meaney, 1999; Feldman, 2012), and influences child’s ADHD symptoms (Biederman et al., 2002; Landau et al., 2009; Lovic et al., 2011; Russell et al., 2016). Still, not all children exposed to adverse and stressful experiences show signs of ADHD symptoms. This heightens the need for research to focus on individual difference factors, including genetic variation and its interaction with environmental factors, to understand heterogeneity in response to early-life adversity (Caspi et al., 2010).

Over the last two decades, molecular genetic studies have identified a few candidate genes with polymorphisms associated with ADHD symptoms and activation of DA within frontal cortex, with the one of most replicated and important being the Catechol-*O*-methyltransferase (COMT) *Val^158^Met* (rs4680) polymorphism (Sun et al., 2014; Mizuno et al., 2017; Jung et al., 2018; Taylor, 2018). The enzyme *COMT* plays an essential role in the catabolism of extraneuronal DA in glial cells and postsynaptic neurons, mainly in the PFC (Pálmason et al., 2010), and is associated with prefrontally mediated cognition, attention (Egan et al., 2001; Bilder et al., 2002; Goldberg et al., 2003), and social behavior (Kereszturi et al., 2008; Halleland et al., 2009; Millenet et al., 2018). Recent studies have shown that a mutation in the *COMT* gene was associated with decreased gray matter volume in children with ADHD (Shimada et al., 2017), and that *COMT* gene was associated with cortical thickness and surface area abnormalities in children with ADHD (Jung et al., 2018). The *COMT Val^158^Met* polymorphism is characterized by a valine-to-methionine (Val/Met) substitution at codon 158. The *Val*^158^ *COMT* enzyme is thermally more stable than the *Met*^158^ version, and catecholamine breakdown is about 40% more efficient in Val-homozygotes, thus conferring lower synaptic DA levels in PFC (Chen et al., 2004), and increased cognitive flexibility, self-regulation and emotion processing (Bishop et al., 2006; Colzato et al., 2010). Met carriers, on the other hand, have greater DA availability in the PFC and limbic areas (Egan et al., 2001), leading to increased high-frequency, low-amplitude tonic DA firing (Bilder et al., 2004). Such an increase affords enhanced stability of cortical activation states, which may lead to the excessive cognitive rigidity and stability that characterize some people with impulsive and hyperactive behavior. Also, Met-carriers exhibited weaker white matter connections compared to in Val-Val carriers (Hong et al., 2014), suggesting that the methionine-allele of rs4860 is involved in white matter maturation and connectivity (Thomason et al., 2010), which may contribute to symptoms of ADHD. Still, studies examining the associations between *Val^158^Met COMT* polymorphism and ADHD have mainly yielded conflicting findings, with a lack of replication and consistent findings as published by recent meta-analyses (Sun et al., 2014; Lee and Song, 2018). These inconsistencies and null findings might be partly due to disregarding the interplay effects of genes with environmental factors, effects which are more likely to be involved in the etiology of ADHD, and may be caused by PFC hypo- and hyper-dopaminergic states when exposed to early-life environmental adversity (Arnsten, 1998; Viggiano et al., 2002).

The mammalian dopaminergic system is known to develop in a dynamic and interactive way in response to a wide range of environmental cues, both positive and negative, early in ontogeny (Gatzke-Kopp, 2011; Feldman, 2017). Therefore, functional variation in *COMT* may contribute to individual DA differences in responses to the environment and may increase the vulnerability of the individual to psychopathologies, such as ADHD. PFC DA has an ‘inverted U’-shaped curve, which means that very high or very low DA levels are associated with reduced synaptic activity in PFC and poor performance for a variety of cognitive, behavioral and executive functions (Arnsten, 1998). Both background PFC dopaminergic tone and individual differences in genes that affect prefrontal DA signaling, act as important modulators of PFC-dependent functions (Dickinson and Elvevåg, 2009). Therefore, the interaction between *COMT* and early-life stressful environmental factors might have a detrimental effect of dopaminergic pathways in the child’s brain, associated with inattention deficits (Voelker et al., 2009; Blair et al., 2015), and impulsivity (Thibodeau et al., 2015; Lovallo et al., 2019). Specifically, against a background of prefrontal hyperdopaminergia under stressful conditions, the slower clearance of DA associated with the methionine variant of the *COMT* gene polymorphism, may exceed optimal levels, with a resultant decrement in PFC functions (Mattay et al., 2003). As such, Met-carriers are more likely to be sensitive to adverse early environments and thus be at a higher risk of developing ADHD. On the contrary, Val-homozygous carriers would be shifted to more optimal DA levels, thereby improving their PFC function, and be at a lower risk of the disorder.

Several studies have supported such GxE interactive pattern by showing that under high psychosocial stress, Met-carriers, compared to Val-homozygotes, showed greater impairments, such as reward hypersensitivity (He et al., 2012), slower executive function development in early childhood (Blair et al., 2015), hypersensitivity to stress, pain and fear induction (Oswald et al., 2004; Jabbi et al., 2007; Armbruster et al., 2012), and higher rates of panic disorder (Asselmann et al., 2018). Still, no study, to our knowledge, has addressed the unique and joint contribution of *COMT Val^158^Met* genetic variation and antecedent family adversity factors, including accumulation SES-risk and negative parenting during infancy, to the development of ADHD across childhood, in a large high-risk sample of children and families living in predominantly low-income and non-urban communities. Two hypotheses were proposed: first, we expected that under high levels of distal and proximal early-life adversity, children with Met allele would show greater ADHD symptom severity, while Val-homozygous children would show less ADHD symptom severity in similar adverse environments (by comparing the two genotype groups under high and low adversity levels). We would not expect to find such differences between groups under low levels of early-life adversity. Second, we expected that children with Met allele would be more sensitive to early adverse environments (show a steeper positive slope) and would exhibit a significant increase in ADHD symptom severity as a function of early-life adversity.

## Materials and Methods

### Participants

The Family Life Project (FLP) was designed to study families (*N* = 1,292) living in two rural areas of high poverty: three counties in North Carolina and three in Pennsylvania (Table 1). Data for this analysis come from data collected in participants’ homes when the target child was approximately 6, 15, 24, and 36 months old. School assessments occurred when the target child was in pre-kindergarten (Pre-k), kindergarten (KG), G1, G2, G3, and G5. Detailed descriptions of participating families and communities are available in Vernon-Feagans et al. (2013).

**TABLE 1:** Demographic and characteristics of the sample by child’s COMT genotype.

**Variable**	**Total**			**COMT Met carriers**			**COMT Val-Val carriers**		
	***N***	**%**		***N***	**%**		***N***	**%**	
*Overall*				604	66.01		311	33.99	
Sex									
Male	457	49.9		304	50.3		153	49.2	
Female	458	50.1		300	49.7		158	50.8	
Race									
African American	514	56.2		393	65.1		121	38.9	
White	401	43.8		211	34.9		190	61.1	
Primary-caregiver COMT Genotype									
Met carriers	546	66.5		445	82.0		101	36.3	
Val-Val carriers	275	33.5		98	18.0		177	63.7	

	***N***	**Mean**	***SD***	***N***	**Mean**	***SD***	***N***	**Mean**	***SD***

Early SES-risk	1235	0	0.69	604	−0.02	0.67	311	0.08	0.67
Early negative caregiving	1221	0	0.59	604	−0.02	0.57	309	0.05	0.60

### Procedure

**FIGURE 1:**
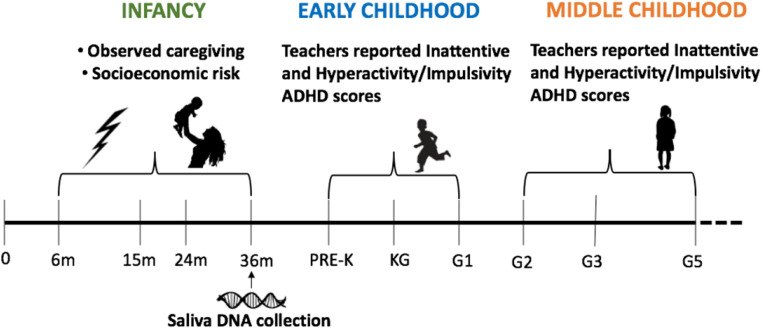
Study design and timeline.

The study design included four visits with each family and six teacher-ADHD evaluations for each child (Figure 1). Families were visited in their homes for data collection twice at child ages 6, 24, and 36 months and once at 15 months. Children’s primary-caregiver, in almost all cases the mother, answered questions about household and demographic characteristics. At 24 months visit, primary caregivers completed the self-report history of ADHD questionnaire. During home visits (6–36 months) primary-caregivers and children participated in 10-min semi-structured interactions. Specifically, at 6 and 15 months, primary-caregivers and infants engaged in a free-play task where primary-caregivers were given a standard set of toys and asked to play as they typically would. At 24 and 36 months, primary-caregivers and infants participated in a puzzle task. Interactions were videotaped and later coded for caregiving behavior. At the home visit for data collection at 36 months, saliva samples were collected using Oragene DNA Self-Collection kits (DNA Genotek, Ottawa, ON, Canada) in accordance with the manufacturer instructions. The current study made use of teachers’ ratings of inattention and hyperactivity/impulsivity ADHD symptom severity when target children were in pre-K, kindergarten (KG), G1, G2, G3, and G5. This study was reviewed and approved by the Institutional Review Board at Pennsylvania State University and the Office of Human Research Ethics at the University of North Carolina.

### Measures

#### Socioeconomic (SES)-Risk

Socioeconomic-risk was represented by computing a cumulative risk composite at 6, 15, 24, and 36 months from seven variables. These variables included family income-to-needs ratio, household density, neighborhood safety, maternal education, a consistent partnership of a spouse/partner living in the home, maximum work hours of primary or secondary caregiver per week, and job prestige. Job prestige was coded using the National Opinion Research Center (NORC) coding system (Nakao and Treas, 1994). To create the continuous cumulative risk index, positively framed indicators were reverse-scored, and each risk measure was standardized and averaged together, such that higher scores indicated higher risk (α = 0.82). As previously reported, this cumulative risk index was originally created through the assessment of nine social risk factors that were demographic indicators of socioeconomic status or had been related to poverty in prior research (Vernon-Feagans et al., 2013). These nine factors included family income-to-needs ratio, household density, neighborhood safety, maternal education, consistently partnered parents, employment hours, and job prestige, as well as maternal health, and ratings of food insufficiency. Principle component analyses at each age revealed a single dominant factor accounting for 33–36% of the total variance among the nine indicators (Vernon-Feagans et al., 2013). With the exception of maternal health and ratings of food insufficiency, all indicators loaded on this factor. As an additional strategy, a within-family mean score was also computed from the repeated measures of each of the nine risk variables from the 6, 15, 24, and 36 months visits. These across-time mean risk scores were moderately correlated with one another, with the exception of maternal health and ratings of food insufficiency. Furthermore, a principle components analysis of these across-time mean risk scores revealed a single dominant factor accounting for 39% of total variance among the nine indicators. Again, the same seven indicators loaded substantially on this main risk index (income-to-needs ratio, household density, neighborhood safety, maternal education, consistently partnered parents, employment hours, and job prestige) (Vernon-Feagans et al., 2013). Both strategies of analyses support that early poverty-related risk can be represented by a single summary variable computed using repeated measures of risk indicators. For the present analyses, a continuous socioeconomic risk index was calculated by reverse scoring positively framed indicators, standardizing each measure, and averaging the standardized values. SES-risk scores were generated for each age (6–36 months), with higher scores indicating higher levels of risk. Scores were averaged to create indices of early SES-risk during the first 3 years of a child’s life.

#### Positive and Negative Caregiving

Global ratings of primary-caregiver behavior were made based on a scale adapted by Cox and Crnic (1999) from the NICHD Early Child Care Research Network (1999) (Blair et al., 2008; Vernon-Feagans et al., 2013). Videos of the target children’s interactions with the primary-caregivers were rated at each time point on seven scales: sensitivity/supportive presence, detachment, intrusiveness, stimulation, positive regard, negative regard, and animation in interacting with the child (NICHD ECCRN, 1999). Ratings for each code were made on a scale ranging from 1 (not at all characteristic) to 7 (highly characteristic). Each scale was coded by a team of 4–5 coders, which included 1–2 master coders. Each coder was trained to be reliable with the master coder(s), and each coder completed approximately 30% of the tapes with the master coder(s). An exploratory factor analysis with an oblique rotation (i.e., Promax) was calculated to inform the development of composite measures of parenting. For consistency, scores at the 24 and 36-months visit were rescaled to range from 1 to 5 (Mills-Koonce et al., 2011). Factor analysis of the caregiving variables yielded two dimensions of caregiving behavior from 6 to 36 months (Mills-Koonce et al., 2011; Vernon-Feagans et al., 2013). Based on those results, we utilized a composite measure of negative caregiving, used as a predictor in this study, which included two characteristics: intrusiveness and negative regard. The second composite, positive caregiving, included sensitivity, detachment (reversed), stimulation, positive regard, and animation codes. Across these four assessments, standardized scores were averaged to create indices of early positive and negative caregiving during infancy. Reliability was determined by calculating intraclass correlation coefficients (ICC) for ratings made by two trained coders for each dimension. ICCs for all dimensions of caregiving ranged from 0.75 to 0.90 across the four time points. Given that positive and negative caregiving were coded as two independent scales and were only moderately correlated at −0.45, negative caregiving in represents more than a *lack* of positive caregiving. Therefore, we include positive caregiving as a control in our model.

#### Genotype

DNA was extracted according to the manufacturer protocol. COMT genotyping was conducted with the appropriate probes for a Taqman SNP Genotyping Assay using an Allelic Discrimination Assay protocol (Applied Biosystems, Foster City, CA, United States). Forty nanograms of DNA were combined in a volume of 5 ml with 2X Universal PCR Mix (Applied Biosystems) and 1/20 the volume of the Taqman SNP assay in a 384 well plate. A Pre-Read was performed and then PCR as follows: a 10 min hold at 95°C, followed by 40–45 cycles of 15 s at 92°C and then 1 min at 60°C in a 7900HT PCR System. After amplification, a Post-Read was performed to analyze. Automatic and manual calls were made (Haberstick and Smolen, 2005). SNPs were quality controlled using procedures outlined previously (Simmons et al., 2010); briefly, quality control required Hardy-Weinberg equilibrium testing *p* < 0.001, missingness by marker <5%, missingness by sample <5%, affirmative relationship checking in PEDCHECK, and Mendelian inconsistency caused genotypes to be dropped at that locus. In the current study, the sample was divided according to the presence or absence of the rare Met allele, by comparing rs4680 Val/Val homozygotes with pooled heterozygote and homozygote carriers of the Met allele (Met-Val and Met-Met). This approach was based on the dominant genetic model, as recommended by previous studies (e.g., van Ijzendoorn and Bakermans-Kranenburg, 2015; Zhang et al., 2016), and was justified by previous observations in healthy and ADHD subjects showing, for example:

(I) Compared with the Val/Val homozygotes, the Met-allele carriers (Met-Met and Met-Val) tend to have one-fourth less COMT enzymatic activity (Lachman et al., 1996) and are hypothesized to exhibit higher dopamine transmission in the prefrontal cortex (PFC) and striatum and no significant differences among Met homozygotes and heterozygotes were found (Chen et al., 2004). A greater hypothalamic–pituitary–adrenal (HPA) axis stress response that correlates with average basal noradrenaline activity has also been found in the Met-allele carriers (Walder et al., 2010).
(II) Met-carriers exhibited smaller striatal GMV than the Val-Val genotype (Shimada et al., 2017, p. 2), while Val-Val ADHD-diagnosed children had significantly lower fractional anisotropy and higher radial diffusivity in the cingulate gyrus compared to ADHD-diagnosed Met-carriers (Basay et al., 2016). Also, white matter connections in Met-allele carriers were weaker than those in Val-Val-allele carriers (Hong et al., 2014).
(III) Distinct associations between Met-carriers and Val-Val-carriers and social impairment, ADHD and other psychiatric disorders (e.g., Barr et al., 2001; Caspi et al., 2008; Sun et al., 2014; Millenet et al., 2018).
(IV) ADHD children who were Val-Val-carriers had significantly better sustained attention (Bellgrove et al., 2005) and cognitive flexibility (Colzato et al., 2014), compared to children with at least one copy of the Met variant.

#### Child Inattention and Hyperactivity/Impulsivity DSM-IV ADHD Symptoms Ratings

Teachers completed an ADHD rating-scale at each grade-visit (Nolan et al., 2001) and rated child’s *DSM-IV* ADHD symptom severity over the last 6 months. The instructions were as following: “*We would like some information about this child over the course of the LAST 6 MONTHS. Please tell us how often this child does the following:*”. All 18 *DSM- IV* symptoms for ADHD were rated on a 4-point Likert-like scale (0 = not at all, 1 = just a little, 2 = pretty much, and 3 = very much). In this study, we focused on separate inattentive and hyperactive/impulsive ADHD factors scores. A mean of inattention ADHD score and a mean of hyperactivity/impulsivity ADHD scores were created separately. Teacher’s inattention and hyperactivity-impulsivity ADHD scores served as the two primary outcomes (Supplementary Table S1). Also, since it has been shown that the PFC undergoes considerable maturation during childhood (Tsujimoto, 2008), including dramatic changes in the DA system during the transition from childhood to early adolescence (Gee et al., 2013), we were interested in investigating the interactive contribution of COMT and early-life adversity on the ADHD symptoms in early (Pre-K - 1st grade) and middle childhood (2nd – 5th grade), separately.

#### Primary-Caregiver’s History of Inattention and Hyperactivity-Impulsivity DSM-IV ADHD Symptoms

At 24-month visit the child’s primary-caregiver completed an ADHD retrospective questionnaire, rating the presence of their *DSM-IV*ADHD symptom severity between ages 5 and 12 on a scale of 0 (not at all) to 3 (very much). The instructions were: “*Read each statement and rate how much it described your behavior when you were between 5 and 12 years of age:*”. A mean of retrospective report of inattentive behaviors, and a mean retrospective report of hyperactive-impulsive behaviors were created separately. Primary-caregivers’ inattentive and hyperactive/impulsive ADHD scores served as covariates in the current study (Supplementary Table S1).

### Data Analysis

To address the hypothesis of gene-by-environment interaction, we ran two multilevel growth curve models (Singer and Willett, 2003), one for each ADHD dimension outcome. Within each model, main effects for *COMT* group (dichotomized: Val-Val vs. Met-Met/Met-Val) and early environmental risk factors (negative caregiving, SES-risk), along with interactions capturing the moderation of each environment variable with the gene were evaluated. Controls for: race, sex, state, age, the primary-caregiver’s ADHD symptoms, and the positive caregiving composite were included in all models. It may well be that primary-caregivers with a specific genetic variant of COMT have an increased probability to use maladaptive caregiving strategies or of being exposed to unstable and risky environment because they have a genetic disposition toward inattention and/or impulsivity, and in such a way transmit a genetic risk for ADHD on to their children (i.e., passive rGE). Since primary-caregivers’ genotype might affect the relation between early-life family environment and children’s ADHD symptoms (Chhangur et al., 2015), by adopting the Keller (2014) approach, we accounted for the passive genetic effect of primary-caregivers’ COMT genotype. We also accounted for other passive genetic effects, such as the primary-caregiver’s genotype × negative caregiving/SES and the primary-caregiver’s genotype × child’s genotype (not shown), that might provide an alternative account of moderating effects of children’s COMT genotype.

#### Model Specification

In these multilevel models, growth was modeled as a quadratic in age. Random effects for the intercept, linear slope and quadratic change over time were chosen in models for both outcomes using the AIC (Akaike, 1974; Burnham and Anderson, 2002). All three covariance terms were included and justified by AIC. Differences in level, indicated by the SD in random intercepts, was 0.7 for both outcomes, which can be compared to effect sizes for other covariates. Intraclass correlations based on the intercept between-subject variance are about 50%, which is quite common in growth curve data. We initially considered a joint model for the two-dimensions of ADHD, which are marginally correlated within person. We determined that the functional form – the impact of covariates on each ADHD subscale outcome – differed substantially, essentially requiring a fully interacted model. In separate models for each ADHD dimension, interactions with *COMT* and time period were considered, and AIC was used to limit the number of interactions to those deemed essential. To keep reporting of the models manageable, we organized the estimated effects into common and period-specific versions in our tables and used standard linear model contrast theory (i.e., Wald tests) to construct standard errors and P-values.

#### Missing Data

To control for potential bias due to missing data, we use multiple imputation (MI; Little and Rubin, 2019) methods, which is equivalent to Full Information Maximum Likelihood (FIML) estimation. 16% of children were never assigned an outcome by a teacher, but we were still able to use the information collected from them in our imputation models. *COMT* genotype was missing on about 30% of children in our study. Instead of deleting cases, we used a Chained Equations (MICE; Van Buuren and Oudshoorn, 1999) approach to impute 100 completed datasets and Rubin’s rules for all subsequent inference (*p*-values, confidence intervals, Wald tests for linear contrasts). Note that both FIML and MI-based approaches relied on the assumption that missingness is “at random” (MAR; Little and Rubin, 2019). For example, this translates to children with *COMT* = Val-Val are not more likely to be missing on the gene sample nor other outcomes. There is no direct test for MAR, so we must consider comparisons of cases under different ways of dealing with missing values and related robustness checks. Comparing measures missing under listwise deletion of cases versus taking all measures (when available), missing subjects scored lower on key variables such as ADHD symptoms and environmental risks (e.g., 0.80 vs. 0.50 for fifth-grade ADHD Inattention). However, after imputation, all the analysis samples had means much more closely aligned with the available case means.

Chi-square tests showed no associations between the groups - participants with no *COMT* data due to loss of follow-up/those with data and categorical variables (see Supplementary Table S2). Negative caregiving scores were essentially identical between the not missing and missing COMT groups, while those missing the *COMT* measure had slightly higher, but not significant, SES-risk scores (a difference of 0.07 SD; *P* > 0.05) and slightly lower, but not significant positive caregiving scores (a difference of 0.12 SD; *P* > 0.05). When we observed outcomes, we saw no difference in the distribution of ADHD symptoms for those missing *COMT* or not. We performed a further set of robustness checks. While it is common procedure to multiply impute all missing predictors and all outcomes of interest (Little and Rubin, 2019), concerns about imputing a gene, given its importance to the research questions, behoove us to compare our stated results with simpler models. We also examined the results of models using only complete cases (at the person-period level, so list-wise deletion in the longitudinal setting) and those that imputed only the predictors and not the COMT (gene) status. Using an alpha level of 0.05, for ADHD Inattention and Hyperactivity symptoms, the results presented here identically significant across both alternative models. For ADHD Hyperactivity symptoms, the results presented here are nearly identical with respect to significance, as does the early childhood interaction of gene with cumulative risk. Thus, we can conclude that our results are consistent with two approaches that rely less on the imputation model, or particularly the model for the gene, further strengthening our confidence in the findings.

#### Protecting Against False Discovery

We also considered controlling for false discovery (FDR) in the eight reported effects using the work of Benjamini and Hochberg (1995), which inflates the *p*-values in a manner similar to Bonferroni adjustment but is more calibrated. Given the number of comparisons and the adjustment, we would expect three of these four effects to remain significant in future, similar studies.

#### Regions of Significance (Probing Moderators)

**FIGURE 2:**
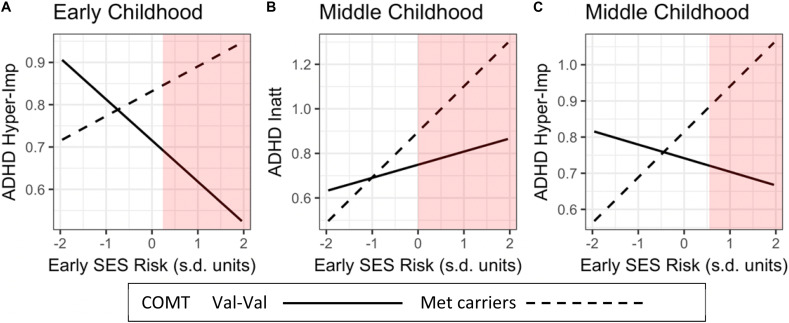
Influences of *COMT* genotype (Met-Met/Met-Val vs. Val-Val)-by-Early life adversity interactions on child’s inattentive and hyperactivity/impulsivity ADHD symptom severity across childhood. The red areas represent the Regions of Significance (RoS). RoS fall within the mean of +2 SD of the independent variable. ADHD Hyper-Imp, ADHD hyperactivity/Impulsivity; ADHD Inatt, ADHD Inattention.

Following Kochanska et al. (2011) and Roisman et al. (2012), we conducted a region of significance (RoS) test (Bauer and Curran, 2005). This technique examines the values of a risk factor (early adversity) for which the moderator (*COMT* genotype) and outcome variable (ADHD symptom severity) are significantly related (at a 0.05 level). The range presented covers 95% of the observed environmental factor (±2 SD). In Figure 2, RoS were calculated for the three significant GxE interactions, using all relevant main and interaction effects estimated in our models.

## Results

### Descriptive Statistics and Measures of Associations

Pearson *X*^2^ statistics were used to analyze the association between child *COMT* genotype and other categorical variables (race and sex), and multiple regression analyses were used to examine the association between genotype and continuous variables (SES-risk and negative caregiving). Frequencies for *COMT Val^158^Met* genotype are reported in Table 1 and were as follows: 604 Met (300 females; 393 African American), 311 Val–Val (158 females; 121 African American) carriers. Columns percents for categorical variables are given in Table 1 for a simpler comparison. *COMT* genotype was unrelated to sex, *X*^2^(df = 1) = 0.065, *P* = 0.798, but was associated with race, *X*^2^(df = 1) = 56.007, *P* < 0.001. African American race was associated with greater odds of having Val-Val genotype, OR = 2.92, 95% CI [2.20, 3.88].

To test for the presence of Gene–Environment correlation (rGE), we examined *COMT* as a predictor of SES-risk scores and negative caregiving scores using multiple regression analysis with controls for race, state and sex. There was no overall association between *COMT* genotype and SES-risk, beta = −0.05, *P* = 0.23, and no overall association between *COMT* genotype and early negative caregiving, beta = −0.06, *P* = 0.11. Descriptive statistics for primary study variables and corresponding bivariate associations appear in Supplementary Table S1 and Supplementary Figure S1, respectively.

### Multilevel Regression Results

#### Outcome 1: Hyperactivity/Impulsivity Symptom Severity

As seen in Table 2, the main effect of *COMT* was not significant in either period. The main effect for negative caregiving was significant for both early and middle childhood, with higher levels of negative caregiving during infancy predicting greater hyperactivity/impulsivity symptom severity. The main effects for SES-risk were not significant in either period. The interactions between *COMT* and SES-risk were significant in both early and middle childhood. The interaction between *COMT* and negative caregiving was significant only in middle childhood but not in early childhood. However, this effect was not identified in our preliminary listwise-deletion-based model and isolated to specific levels of SES-risk identified in exploratory analyses. We took this to indicate that it was not robustly supported in our data.

**TABLE 2:** Multilevel growth curve models predicting child’s hyperactivity/impulsivity ADHD symptom severity over time.

	**Estimate (effect size)**	**Standard Error**	***P*-value**	**Sig.**
**Common**				
State (PA = 0, NC = 1)	−0.005	0.053	0.922	
Sex (Female = 0, Male = 1)	0.348	0.042	0.000	***
Race (White = 0, African American = 1)	0.151	0.058	0.009	**
Age (cntrd)	−0.006	0.012	0.596	
Age Squared	−0.011	0.003	0.001	***
Primary-caregiver COMT (Met = 0/Val-Val = 1)	0.068	0.056	0.224	
Primary-caregiver ADHDHyper-Imp	0.013	0.032	0.680	
Primary-caregiver ADHDInatt	−0.001	0.034	0.983	
Positive caregiving	−0.066	0.028	0.017	*
**Early childhood**				
(Intercept)	0.832	0.043	0.000	***
COMT (Met = 0/Val-Val = 1)	−0.116	0.072	0.108	
Negative caregiving	0.115	0.029	0.000	***
SES-risk	−0.019	0.032	0.550	
Negative caregiving × COMT	−0.004	0.063	0.955	
SES-risk × COMT	−0.156	0.070	0.025	*
**Middle childhood**				
(Intercept)	0.815	0.039	0.000	***
COMT (Met = 0/Val-Val = 1)	−0.073	0.074	0.323	
Negative caregiving	0.135	0.030	0.000	***
SES-risk	0.044	0.033	0.179	
Negative caregiving × COMT	0.147	0.062	0.042	*
SES-risk × COMT	−0.164	0.069	0.018	*

Multilevel regression results: estimated effect sizes are organized into common, early and middle childhood. Variance components are not given. See text for details. * *P* < 0.05, ** *P* < 0.01, *** *P* < 0.001.

#### Outcome 2: Inattention Symptom Severity

As seen in Table 3, the main effect of *COMT* was not significant in early childhood but significant in middle childhood, showing that children carrying Met allele had higher levels of inattention severity symptoms compared to Val-Val carriers. The main effect sizes for negative caregiving in both early and middle childhood were significant, with higher levels of negative caregiving during infancy predicting higher inattention symptom severity. The main effect sizes for SES-risk were significant in early and middle childhood, suggesting that higher SES-risk during infancy predicted greater inattention symptom severity. The interactions between *COMT* and negative caregiving were found non-significant in both time-periods. The interaction between *COMT* and SES-risk was found significant only in middle childhood but not significant in early childhood, even though a similar pattern of interaction was found.

**TABLE 3:** Multilevel growth curve models predicting child’s inattentive ADHD symptom severity over time.

	**Estimate (effect size)**	**Standard error**	***P*-value**	**Sig.**
**Common**				
State (PA = 0, NC = 1)	−0.051	0.050	0.308	
Sex (Female = 0, Male = 1)	0.342	0.041	0.000	***
Race	0.100	0.055	0.071	
Age (cntrd)	0.072	0.012	0.000	***
Age squared	−0.022	0.003	0.000	***
Primary-caregiver COMT (Met = 0/Val-Val = 1)	0.006	0.052	0.910	
Primary-caregiver ADHDHyper-Imp	0.027	0.029	0.362	
Primary-caregiver ADHDInatt	−0.008	0.033	0.819	
Positive caregiving	−0.090	0.027	0.001	***
**Early childhood**				
(Intercept)	0.972	0.042	0.000	***
COMT (Met = 0/Val-Val = 1)	−0.071	0.069	0.298	
Negative caregiving	0.088	0.027	0.001	**
SES-risk	0.060	0.030	0.047	*
Negative caregiving × COMT	−0.078	0.057	0.173	
SES-risk × COMT	−0.064	0.061	0.296	
**Middle childhood**				
(Intercept)	0.897	0.038	0.000	***
COMT (Met = 0/Val-Val = 1)	−0.148	0.073	0.043	*
Negative caregiving	0.090	0.030	0.003	**
SES-risk	0.132	0.032	0.000	***
Negative caregiving × COMT	0.069	0.062	0.266	
SES-risk × COMT	−0.146	0.066	0.026	*

Multilevel regression results: estimated effect sizes are organized into common, early and middle childhood. Variance components are not given. See text for details. * *P* < 0.05, ** *P* < 0.01, *** *P* < 0.001.

### Region of Significance Analyses

To illuminate the nature of the four significant GxE interactions, we plotted regression slopes of early-life adversity (SES-risk and negative caregiving) on ADHD symptom severity separately for children with Val-Val genotype and at least one Met allele (Figures 2A–C). Next, we conducted a region of significance (RoS) test to determine whether the GxE interactions support the diathesis-stress or the differential-susceptibility model (Bakermans-Kranenburg and Van IJzendoorn, 2007; Roisman et al., 2012). In Figure 2A, RoS analysis revealed that in early childhood, children with Met alleles had significantly higher levels of hyperactivity/impulsivity symptom severity than children with Val-Val, only when exposed to higher than the average levels of SES-risk during infancy. In Figure 2B, RoS analysis revealed that in middle childhood, children carrying Met allele had significantly higher levels of inattention symptom severity than children carrying Val-Val alleles, only when exposed to higher than the average levels of SES-risk during infancy. In Figure 2C, it revealed that in middle childhood, Met-carriers had significantly higher levels of ADHD hyperactivity-impulsivity symptom severity than children with Val-Val, only when exposed to higher levels of early SES-risk (i.e., above +0.50 SD).

In addition, we conducted simple slopes analysis. In Figure 2A, the effects of SES-risk on ADHD-hyperactivity/impulsivity in early childhood did not reach significance for Met carriers (*P* = 0.13), and Val-Val carriers (*P* = 0.08). In Panel B, the effects of SES-risk on ADHD-Inattention in middle childhood was significant only for Met carriers (*P* = 0.003) but not for Val-Val carriers (*P* = 0.26) In panel C, the effects of SES-risk on ADHD-Hyperactivity/impulsivity in middle childhood was significant only for Met carriers (*P* = 0.007) but not for Val-Val carriers (*P* = 0.49).

## Discussion

Our large longitudinal birth cohort study provides a unique opportunity to detail the implications of the interplay between observational and self-reported familial environmental risk factors during infancy and *COMT* gene *Val^158^Met* polymorphism on ADHD symptoms across childhood in population sample of low-income children. Our findings show that both proximal factors, such as the quality of caregiving, and more distal ones, such as family income, caregivers’ education, neighborhood safety and household density, are related to developing childhood ADHD symptoms, as reported by child’s teachers. Previous studies have shown that teacher rating made a stronger contribution to the prediction of ADHD dimentions than parent ratings (Power et al., 1998). Still, while negative caregiving in infancy, regardless of child’s genotype, showed an increased risk for inattention and impulsivity/hyperactivity symptoms across childhood, SES-risk interacted with child’s *COMT* genotype in predicting ADHD symptoms across childhood.

To our knowledge, the present study is also the first to examine similarities and differences in the genetic, cumulative environmental and GxE effects on the two ADHD dimensions in early childhood – when the transition to school places new demands on the child’s self-regulation and attention skills, and in middle childhood - when substantial changes in multiple features of child’s life, including psychological and neuroendocrine changes, occur (Tseng and O’Donnell, 2006). We found an association between child’s *COMT* and inattention symptom severity in middle childhood, but not in early childhood, and not with impulsivity/hyperactivity symptoms. Results agree with those of most recent meta-analyses (Sun et al., 2014; Lee and Song, 2018), showing an absence and inconsistency of association between *COMT* and ADHD, leading us to conclude that ADHD is not a unitary disorder and in fact different alleles contribute to different domains of ADHD-related symptoms across development.

Decades of empirical and theoretical research have provided evidence that early environment exerts a high level of influence on child development (Shonkoff and Phillips, 2000). More specifically, the first 3 years of a child’s life are increasingly recognized as a critical period for brain growth and a window of opportunity to optimize children’s social-emotional development and cognitive functioning (Groh et al., 2012). We found that negative caregiving in infancy independently predicted a child’s ADHD symptom severity across childhood, and SES-risk predicted inattention symptom severity across childhood, but not hyperactivity/impulsivity symptoms. Our findings are supported by animal studies showing that adverse environmental conditions and reprogramming of HPA axis impact the development of the mesocortical and mesolimbic DA system in limbic regions and the PFC (Tost et al., 2015), and by human studies reporting that exposure to early adversity during the first years of life elevates the likelihood of poor neurobehavioral development, impairs attention and self-regulation skills, and increases the risk for mental disorders (Yoshikawa et al., 2012; Blair and Raver, 2016; Perry et al., 2018).

Turning to GxE interactions, our hypotheses were partially supported. As expected, we found *COMT*-by-SES-risk interactions for hyperactivity/impulsivity phenotypes across childhood and for inattention in middle childhood. RoS and simple slope analyses confirmed our hypotheses. Met allele-carriers’ positive slopes indicated that these children were more vulnerable to an early-life negative environment and exhibited a significant increase in ADHD inattention and hyperactivity/impulsivity symptom severity in middle childhood as a function of early-life SES-risk. Met carriers also exhibited significantly greater ADHD symptom severity in early and middle childhood compared to Val-Val carriers, when exposed to high SES-risk levels. No differences between allele groups were found under low SES-risk levels. Val-Val carriers had negative or flatter positive slopes which not reach significance, indicating the Val-Val carriers did not show any increase in ADHD hyperactivity/impulsivity symptom severity in early childhood as a function of SES-risk. Such differences under stressful conditions, possibly due to PFC hypo- and hyperdopaminergic states, have previously been suggested as part of the “warrior-worrier” model (Enoch et al., 2006). In this model, among Met-carriers, adversity may increase levels of DA beyond effectual levels which may result in worse performance. Moreover, only one *COMT*-by-negative caregiving interaction was found, but it was not found in listwise-deletion-based model, was not consistent across ages and ADHD dimensions, and found to be driven mainly by different levels of SES-risk in our exploratory analyses. Therefore, it should be interpreted with caution and requires replication before drawing definitive conclusions. While a few previous studies pointed to different effects of types of life stress on the mesocortical DA functioning (Sullivan and Gratton, 1998), it still remains unclear why variations in the *COMT* gene interact exclusively with early-life SES-risk or differently than with negative caregiving in predicting ADHD symptoms.

Chief among the strengths of the present study are the genetically informed longitudinal design with its sophisticated analytic strategy. Still, several study limitations should be considered. First, since our sample is predominantly high-risk and low-income, implications of results may not be extendable to other socioeconomic and ethnic groups with different allele frequencies or environmental/social factors, which may impinge differently upon ADHD symptoms. Future studies should compare group of children from high-risk families to those from better-off families on the distribution of COMT genotypes and the occurrence of ADHD. Second, for a candidate gene study, our sample is relatively small to further subdivide the sample to perform a replication analysis. Future studies should use a much larger sample sizes for population-based genetic association studies. Third, we examined the effect of only a single genetic variant on ADHD. Although the *COMT Val^158^Met* polymorphism is widely studied in relation to ADHD (Thapar et al., 2005; Caspi et al., 2008), future studies aiming to probe the effect of gene-by-early adversity on ADHD symptoms should expand the focus on other candidate genes or polygenic risk scores, including other DA system genes, serotonin system genes, adrenergic genes, and cholinergic genes. Finally, although the present study further supports the role of gene-by-early life adverse family environment interaction in the etiology of ADHD, our results provide no evidence for the exact neuronal mechanism by which this GxE interaction confers risk for the disorder.

## Conclusion

Overall, the present study lends further support for the complex interplay of genetic differences and environmental factors in the etiology of childhood ADHD. The present study provides the first evidence for the longitudinal mutual influence of *COMT Val^158^Met* genotype and early-life family adversity on childhood ADHD symptoms in a population sample of low-income children, with effects of early-life environment on ADHD risk were most pronounced in Met carriers. Our findings highlight the need to identify genetic and environmental risk factors during first years of life, to monitor those who are at risk for exhibiting elevated ADHD symptomatology, and to construct individually tailored early-life family system and caregiver-child interventions.

## Data Availability Statement

The datasets presented in this article are not readily available because they are not publically available. Requests to access the datasets should be directed to EA, Eyal.abraham@nyspi.columbia.edu.

## Ethics Statement

The studies involving human participants were reviewed and approved by the Institutional Review Board at Pennsylvania State University and the Office of Human Research Ethics at the University of North Carolina. Written informed consent to participate in this study was provided by the participants’ legal guardian/next of kin. Written informed consent was obtained from the individual(s), and minor(s)’ legal guardian/next of kin, for the publication of any potentially identifiable images or data included in this manuscript.

## Author Contributions

EA and CB contributed to the conception and design of the study. EA and MS organized the database and performed the statistical analysis. EA wrote the first draft of the manuscript. MS and CB wrote sections of the manuscript. All the authors contributed to the manuscript revision, read and approved the submitted version.

## Conflict of Interest

The authors declare that the research was conducted in the absence of any commercial or financial relationships that could be construed as a potential conflict of interest.

## Supplementary Material

TABLE S1 Descriptive statistics for observed variables.

TABLE S2 Comparison of Covariates for NOT missing and missing child’s genotype data.

FIGURE S1 Correlation plot between primary variables at each time point. Sign of the correlation is color-coded (red negative; blue positive) and correlations deemed non-significant at the 0.05 level are overwritten with a large ‘X’. ADHD HyImp, ADHD hyperactivity/Impulsivity; ADHD Inatt, ADHD Inattention.
