# Supplementary material for: Catechol-O-methyltransferase Val158Met Genotype and Early-Life Family Adversity Interactively Affect Attention-Deficit Hyperactivity Symptoms Across Childhood
Source: Front Genet. 2020 Jul 10;11:724. doi: 10.3389/fgene.2020.00724 (PMC7381281; doi:10.3389/fgene.2020.00724)
Supplement: TABLE S1 — Descriptive statistics for observed variables. [file Data_Sheet_1.docx]

**Supporting Information:**

**Table S1- Descriptive statistics for observed variables**

| **Variable** | **N** | **Mean** | **SD** | **Min.** | **Max.** |
| --- | --- | --- | --- | --- | --- |
| Early SES-risk | 1235 | 0.00 | 0.69 | -2.66 | 2.19 |
| Early observed negative parenting | 1221 | 0.00 | 0.59 | -1.35 | 2.15 |
| Early observed positive parenting | 1221 | 0.00 | 0.66 | -1.85 | 1.80 |
| ADHD Inatt Pre-K | 816 | 0.57 | 0.64 | 0 | 3 |
| ADHD Inatt Kindergarten | 984 | 0.74 | 0.81 | 0 | 3 |
| ADHD Inatt Grade 1 | 929 | 0.78 | 0.84 | 0 | 3 |
| ADHD Inatt Grade 2 | 928 | 0.81 | 0.83 | 0 | 3 |
| ADHD Inatt Grade 3 | 848 | 0.81 | 0.79 | 0 | 3 |
| ADHD Inatt Grade 5 | 703 | 0.76 | 0.79 | 0 | 3 |
| ADHD Hyper-Imp Pre-K | 816 | 0.62 | 0.68 | 0 | 3 |
| ADHD Hyper-Imp Kindergarten | 984 | 0.64 | 0.76 | 0 | 3 |
| ADHD Hyper-Imp Grade 1 | 929 | 0.67 | 0.80 | 0 | 3 |
| ADHD Hyper-Imp Grade 2 | 928 | 0.65 | 0.76 | 0 | 3 |
| ADHD Hyper-Imp Grade 3 | 848 | 0.59 | 0.72 | 0 | 3 |
| ADHD Hyper-Imp Grade 5 | 703 | 0.52 | 0.68 | 0 | 3 |
| Primary-caregiver ADHD Inatt | 1102 | 0.44 | 0.54 | 0 | 3 |
| Primary-caregiver ADHD Hyper-Imp | 1102 | 0.40 | 0.50 | 0 | 3 |

ADHD Hyper-Imp, ADHD hyperactivity/Impulsivity; ADHD Inatt, ADHD Inattention

**Table S2-** Comparison of Covariates for NOT missing and missing child’s genotype data

|  | **N** | **%** | ***COMT***  **NOT**  **missing n** | ***COMT***  **NOT**  **missing: %** | ***COMT***  **missing: n** | ***COMT***  **missing: %** |
| --- | --- | --- | --- | --- | --- | --- |
| **SEX: Male** | 635 | 49.1 | 457 | 49.9 | 178 | 47.2 |
| **SEX: Fem** | 657 | 50.9 | 458 | 50.1 | 199 | 52.8 |
| **RACE: AfAm** | 743 | 57.5 | 514 | 56.2 | 229 | 60.7 |
| **RACE: white** | 549 | 42.5 | 401 | 43.8 | 148 | 39.3 |
| **Primary-caregiver COMT=Met** | 578 | 66.1 | 546 | 66.5 | 32 | 60.4 |
| **Primary-caregiver COMT=Val-Val** | 296 | 33.9 | 275 | 33.5 | 21 | 39.6 |

**Figure S1: Correlation plot between primary variables at each time point**


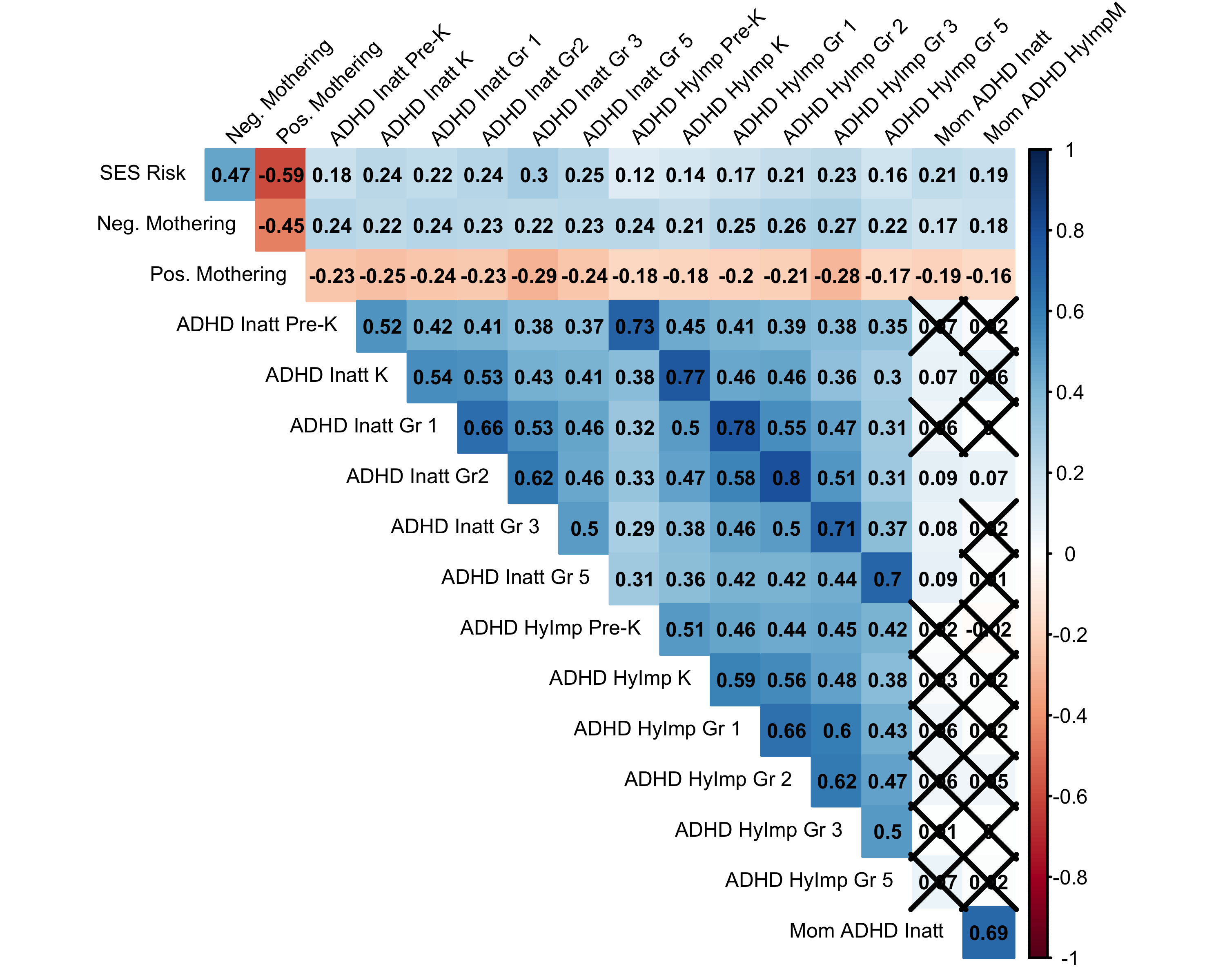


Sign of the correlation is color-coded (red negative; blue positive) and correlations deemed non-significant at the 0.05 level are overwritten with a large ‘X’. ADHD HyImp, ADHD hyperactivity/Impulsivity; ADHD Inatt, ADHD Inattention.
